# The Surgical Treatment of “Dyspepsia”1The annual address in surgery, read before the Wisconsin State Medical Society at Milwaukee, June 23, 1904.

**Published:** 1904-07

**Authors:** Roswell Park

**Affiliations:** Buffalo, N.Y., Professor of Surgery in the University of Buffalo


### BUFFALO MEDICAL JOURNAL.

VOL. XLIII.—LIX. JULY, 1904. No. 12

### ORIGINAL COMMUNICATIONS.

### The Surgical Treatment of "Dyspepsia."[1]

BY ROSWELL PARK, M. D., LL.D., Buffalo, N. Y.,
Professor of Surgery in the University of Buffalo.

THE terms indigestion and dyspepsia,—like the term rheumatism,—are applied so vaguely and made to cover such a variety of conditions, that I have purposely chosen one of them for a heading for this very reason. We may assume that both are meant to include such symptoms as discomfort after eating, nausea, often actual vomiting, pain—often severe, emaciation, loss of appetite, perhaps change in color of the skin, amounting to more or less jaundice, a host of other and of minor symptoms, plus whatever may be discovered on physical examination, or that of the excreta. For each and all of these there must be a cause. Modern methods of diagnosis have taught us how to recognise and deal with many of them, by means quite outside the pale of surgery, and quite within that of dietetic and medicinal treatment. But modern methods have also done much to reveal how many of the conditions, formerly so vaguely grouped and so empirically treated, are, first, impossible of accurate recognition, or, second, impossible of suitable and successful internal therapy, or, third, accurately recognised only when it is very late, or too often too late to repair damage already done when treated by old conventional methods, so that there has sprung up a general feeling of dissatisfaction therewith, with a growing sentiment in favor of direct exploration, with direct attack if the condition revealed thereby seem to justify it.

The internist—as such—has seen, as it were, one organ after another taken away from him or placed under the joint juris-

1. The annual address in surgery, read before the Wisconsin State Medical Society at Milwaukee, June 23, 1904.

diction of the surgeon, until he may almost well feel that scarcely any part of the human body is sacred to the physician or exempt from the surgeon's attack. It is not strange, then, if some evidences of personal feeling creep now and then into discussion of these matters, nor can tradition and custom be instantly set aside when men try to adjust themselves to new relations.

If then one were to begin by saying that ulcer of the stomach and duodenum, cholelithiasis, appendicitis, pancreatitis, and the like, are essentially surgical conditions, to be dealt with by the surgeon, he would be perpetrating an aphorism which he would find many men loath to adopt; while, if he were to insist that cancer of the stomach is a lesion which should be dealt with by the surgeon often long before it is recognised by the physician, he would certainly shock some morbidly sensitive minds, while announcing a great truth. For I believe that cancer is at first a local disease, and that—as I have often said—there is a period in the history of every cancer when if it could be recognised, and if it were accessible, and if it were thoroughly enough removed, it might be cured. But these "ifs" are of tremendous importance, and stand in the way of success in the majority of cases. But by the time cancer of the stomach is really recognised it is usually too late to do anything more than palliate, even by a skilful surgical operation.

But let us assume the condition, which fortunately generally prevails, that practitioners,—whatever their particular bent or tastes,—are honest and sincere men, anxious first of all for the welfare of their patients, ready and willing to advise that which they deem best, needing only to see clearly that in a given case this method or that gives greater promise of success. If to such an one it can be clearly presented that surgical measures offer better prospect than medicinal, then he needs only to be convinced in order to recommend them. To be sure, and at the present day, such conviction may necessarily include the putting aside of all his old teaching and the relinquishment of much that he has long believed and practised. It may also include such surrender of case and fee as may seriously test his absolute sincerity. With that feature of the problem, however, we are not at present concerned, rather only with its scientific merits.

This much by way of introduction. My work as a surgeon specialist has so often brought me in contact with lesions of the upper abdomen, which have proved to be essentially surgical, but which have for months or years been under medicinal care, or even under little or no care, that I have come to view some of these cases almost with dread, fearing that it be too late, or that a given expedient is now desperate which resorted to earlier would have been safe. And I dread, too, the imputation which every now and then some guilty practitioner makes to shield himself: "Well, you had an operation; now see the result;" dread it because our ethical observances do not justify one in retorting, "True, but he died because you advised him against it until it was too late." Over the autopsy table there should be no unseemly wrangling, but the lessons there inculcated should never be lost.

Some of the lessons thus learned I beg to discuss with you on this occasion. And for this discussion let it be confined essentially to four of the contents of the upper abdominal cavity—namely, the stomach, the duodenum, the biliary passages and the pancreas, and to only such lesions of these parts as are now thought by the most progressive, but which will in a comparatively short time be acknowledged by all, to be surgical in their nature; *i. e.,* best or only to be treated by operative methods. I shall confine myself to conditions which are more or less associated with those disturbances so often collectively spoken of as "dyspepsia" and "indigestion." (Hence the title of this paper.)

### THE STOMACH.

*Cardiospasm.*—Not long ago Zenker described a condition of apparently spontaneous dilatation of the esophagus, occurring in middle and elderly life, characterised by difficulty in swallowing which appeared to be caused by an associated spasm of the muscle structures of the cardiac end of the stomach. There results from such spasm more or less cardiac obstruction and esophageal expansion, the lower end of the gullet seems to retain a portion of the ingesta which should find prompt entrance into the stomach. This amount may in time equal a half litre, and patients have even died of inanition without minute inquiry into the condition which causes this. It is a lesion which corresponds to pyloric obstruction and must be dealt with after the same mechanical fashion, though, to be sure, plastic operation has there been substituted for the older dilatation by Loreta's method. Such method is still applied at the cardiac end of the stomach, as Mikulicz and others have shown, and a considerable series of cases now brilliantly illustrates the benefits to be obtained in cardiospasm through surgical operation and through it alone.

*Pyloric Obstruction.*—The question of the possibility of a congenital stenosis of the pyloric is perhaps still under discussion. The best and most recent reports seem to be that such condition is possible, and in 1901 there were some forty examples on record. Regarding the symptoms of this we need not be detained here, yet they show the possibility of such condition as perhaps explaining intractable vomiting in the very young. Operation has been successfully performed on an infant of eight weeks, and there are several successful reports concerning infants not much older than this. Naturally, the operation of choice in any such case would be a posterior gastroenterostomy. In so young a patient this should be performed entirely by the suture method, and no artificial help (*i. e.,* button) should be employed.

*Gastric Ulcer.*—This constitutes a very common and unpleasant, often distressing, and sometimes fatal lesion for which medicinal treatment may be justified early but for which, in serious cases, there is no real cure except that afforded by surgery. It is not intended here to study its causes, but a few words explaining the condition itself would not be amiss. These lesions are usually spoken of as simple erosions, acute round ulcer, and chronic ulcer. The erosions have not had their share of popular attention, because they are often so apparently trifling as to be neglected. They may elude what seems to be a careful search during operation and they are likely to be overlooked during the ordinary post-mortem examination. They may be so small as to be barely perceptible and may assume the type of fissure, and yet from so insignificant a lesion most alarming hemorrhages may result. This gives them an importance quite disproportionate to their size, and I am sure it is not generally appreciated what serious results may occur from so apparently trivial a crack in the mucosa. These lesions are most common in the pyloric end of the stomach, for obvious reasons. The acute or subacute round ulcer is more often found in women, while the chronic form with its irregular outlines and thickened edges is more often seen in men.

Three of the significant symptoms of ulcer are pain, vomiting, and hematemesis. The pain is located in the epigastrium and radiates not infrequently toward the back and region of the left shoulder. It is produced by the ingestion of food. Vomiting takes place usually within an hour after food-taking, and by it the stomach contents are usually completely expelled. Bleeding is much more frequent in the acute than in the chronic type of ulcer. In the slow and insidious cases of chronic ulcer pain may not occur until an hour or two after filling the stomach. Vomiting is irregular, often large in quantity, and the vomitus is often of the coffee-ground type, sometimes fetid; this depends to a considerable extent on the amount of dilatation of the stomach and the type of fermentation of its contents. In the slowest cases the symptoms are very irregular. The most chronic cases are more likely to have pyloric obstruction and dilatation.

In nearly all these cases a great amount of tenderness in the epigastric region and sometimes enlargement and thickening of the pyloric region can be made out, in addition to evidence of dilatation which a careful physical examination will furnish. The pain and tenderness are usually quite well localised, more so than in many other abdominal conditions. An ulcer of the anterior surface of the stomach, near the cardiac end, will cause tenderness along the left costal arch and median line and will be relieved by the horizontal position, while an ulcer of the pyloric end gives tenderness to the right of the median line and the pain is relieved by lying on the left side and increased by lying upon the right. With an ulcer of the posterior wall, the pain is felt in the back and beneath the left shoulder blade. When there are firm adhesions to the liver, the pain radiates under it and in the direction of the right shoulder. When the pain is severe vomiting is rarely absent, and this act usually gives prompt relief. One must not forget that there are also so-called "latent ulcers," which give rise to very few symptoms until something alarming suddenly happens.

While mild cases of suspected gastric ulcer may be amenable to nonoperative treatment a serious and alarming manifestation, such as hemorrhage and intense pain, should be followed by operation at the earliest practical moment. No one can foretell exactly what it will be necessary to do, and so one should be prepared for anything that may be required. The English surgeons, of whom Robson and Moynihan are the most conspicuous in this work, seem to rely almost entirely on gastroenterostomy and do not recommend opening the stomach and searching for the site of the ulcer, but my own experience with this method, though small as compared with theirs, has been so satisfactory that I could not personally advise against an exploration, at least in select cases. The ulcer, if found, may be excised or curetted, and the surrounding area closed with sutures; or there might be found such adhesions to the liver, for instance, as to make it inadvantageous to detach these, but make it wise to proceed at once to anastomosis. On the other hand, unless the adhesions are relieved, the patient will not be freed from the radiating pain toward the right shoulder, of which he has probably sorely complained. A reasonable effort, then, should be made to free the pyloric region from anchorage, and if during the process the stomach or duodenum be opened sutures may be used freely and anastomosis be made for protection. With anastomosis performed, as Moynihan has taught us, in an antero-posterior or vertical direction, so that there shall be left no retaining pouch of the stomach, the relief afforded is complete and, as it appears, final.

Pyloroplasty has been almost abandoned by the gentlemen I have quoted above, and yet, here again, I have seen beautiful results which make me loath to entirely give it up.

The simple suture method without complicated instruments and such aid as the metallic button, and the like, are certainly the most adaptable for this purpose. The opening can be made as large as desired, and there is no necrotic process upon which one has to rely for the success of his operative venture. Of course, all that is said here with regard to gastric ulcer is intended to apply more positively to its complex forms, some of which are serious and some of which are promptly fatal unless skilfully operated. I am dealing rather with the general phase of the subject which leads one to say, speaking as a surgeon, that every case so diagnosticated and every case of suspected gastric ulcer would better be operated providing, of course, the patient can be placed in proper hands.

*Gastric Dilatation.*—Of the extent to which this lesion may go, I do not need remind you. Of course, it is most frequent and excessive in pyloric obstruction or cases of torsion. The greater the amount of dilatation the less hopeful the case from the nonoperative side. Dilatation is to be suspected when there is an increase of thirst with poor appetite and an almost constant feeling of dryness in the throat, when considerable amount of gas is expelled from the stomach, and when these symptoms accompany an almost constant feeling of oppression or soreness in the epigastrium. Vomiting is a feature of the advanced cases and may be a daily or less frequent action, the rejected matter showing evidences of decomposition and free hydrochloric acid. When in such a case, in spite of regular lavage and a properly regulated diet, there is no improvement, then the operation of gastroplication is indicated and will be almost always successful. The process is almost simplicity itself, exposing the patient to very little risk and giving great prospects of relief. It consists in taking a series of tucks in the stomach wall by which its capacity as a retaining bag is very much reduced.

*Gastroptosis* was first described by Glenard in 1885, and is often called by his name. It may occur along with the displacement of other organs. By virtue of its change of position the stomach may become obstructed at its pyloric end and thus a vicious circuit be established from which there is small relief save by operation. Duret was the first surgeon to suggest this relief, which he afforded by suturing the lesser curvature to the abdominal fascia. A gastroplication might also be easily combined with this gastropexy.

*Cancer of the Stomach.*—Obviously, if the surgeon be of much use in such case he must deal with primary cancer and with one not too far advanced. The extraordinary infrequency with which cancer transgresses the pyloric boundary of the stomach is fortunate for the surgeon, but a feature which no one yet has been able to explain. Adhesions and lymphatic involvement are present in at least one half of the cases. Probably five-eighths of a large total of cases of gastric cancer will be found to involve the pyloric region, but cancer may be met with at any point of the stomach wall. It affects males more commonly than females and is ordinarily spoken of as a disease of late middle or advanced life, yet it has been observed in an infant of five weeks and operated upon in the twentieth year of life. These cases vary much in their acuity and are often divided into the acute and latent. So rapid may be the course of such a lesion, in rare instances, as to destroy life within three months from the first recognisable symptoms. On the other hand, they may run an exceptionally long and almost latent course like some cases of cancer of the breast.

Inasmuch as cancer is a disease which has no essential symptomatology, so here it is only as one function or another is disturbed by the advance of disease that there arise either symptoms or signs. As a rule, they do not appear until the disease has made considerable encroachment and consist then of vomiting, pain and presence of a tumor. In the presence of these three it is rarely difficult to make a diagnosis, but by the time they are present it is often too late to accomplish much by surgery. The earliest symptoms are vague and misleading, and include what is ordinarily meant in the popular expression "dyspepsia." There is also included a certain amount of discomfort, with occasional nausea and malassimilation of food, which, of course, means loss of strength and body weight. These symptoms will, of course, depend to some extent on the location of the lesion, this being true also of the gastric ulcer; for instance, the formation of hydrochloric acid and pepsin seems to take place rather in the middle zone of the stomach, and the size and location of the cancer may explain the absence or presence of free hydrochloric acid, upon which so much stress is laid, but which at the same time proves so unreliable in recognising the disease. A cancer located near the pylorus will cause obstruction much earlier than one at a distance. Thus it may happen that obstruction symptoms with dilatation form the most pronounced early indication of gastric disorder. Cancer high up near the cardia will produce few early symptoms. By the time it can be positively recognised the patient is too far advanced in the disease to justify anything more than a palliative operation. According to the degree of dilatation and the movability of the stomach will be the size and location of the tumor when recognised. If adherent to the liver, as it often is, it may be located fairly high up in the abdomen; on the other hand, the stomach may be so stretched out of shape as to carry it below the umbilicus, where it might be mistaken for something else were not lavage or distension intelligently practised. In many respects a dilated and movable stomach may be operated more easily, especially about the pylorus, than a case characterised by firm adhesions, in which case radical operation is rarely of benefit.

After all, the principle matter in these cases is to resort to surgery early rather than late, for which probably we still need a means of early diagnosis which is not yet forthcoming. Almost the best that can be said is that one may suspect the presence of cancer without being able to prove it; and this leads me here to emphasise, and I desire to make the emphasis as strong as possible, that in my opinion a well-founded suspicion of cancer of the stomach, as well as of various other organs, amply justifies an operation intended, first, for its discovery or determination, and, secondly, for its relief. It is hardly worth the time here to go into the chemistry or tests by which this suspicion may be strengthened. I care not by which method the conclusion or the suspicion is arrived at, nor do I care whether the diagnosis wavers as between cancer, ulcer or benign tumor. The rule will apply equally well in the less malignant lesions and happier will the surgeon be in its application.

*Ulcer of the Duodenum.*—Duodenal ulcer has many characteristics similar to those of gastric ulcer, the pain and tenderness being rather vague, with occasional sharp spasms or stitches upon any sudden action, like turning quickly or sneezing. The hemorrhage from this source may escape either through the stomach and mouth or by the bowels, but in no case is it likely to be so fresh as when it takes place from a gastric ulcer. The pain which follows the ingestion of food will not ensue so early, since it is not produced until the food begins to enter the intestine. Moynihan, in 1901, showed how in 18 cases out of a total of 49 a diagnosis of appendicitis had been made and operation undertaken for that condition. This fact cannot be regarded as entirely due to inattention to the possibility of duodenal ulcer, since the complaint of pain is often vague and its reference to the lower part of the abdomen is quite common. Moynihan has also emphasised what I have myself seen,—namely, that in addition to ulceration of either the stomach or duodenum there occur minute erosions, barely recognisable even on close scrutiny, which open up bloodvessels, and that there occur so-called "weeping patches" with various other indeterminate conditions.

When a long train of so-called dyspeptic symptoms is followed by exacerbation with sudden access of pain, more or less faintness or collapse, and especially by vomiting of blood or melæna, and when there is tenderness in the region of the upper abdomen, we may well suspect a duodenal ulcer which has given away. In some respects the extent of the perforative lesion is in proportion to the acuity of the symptoms. That perforation does occur more often than is generally suspected is doubtless the case. If it be minute by a localised peritonitis, in some instances a protective wall is quickly formed which prevents the invasion of the general peritoneal cavity, and leads to adhesions which shut off the diseased area, while they at the same time cement the viscera together. Various operators have reported such adhesions which make it apparent that something of this kind had happened. With a diagnosis of perforating ulcer it would be extremely hazardous to postpone exploration in the hope that this might occur.

When actual perforation does occur the symptoms are overwhelming, and sometimes one is confused by their resemblance to those which are common to several of the acute lesions of the upper abdomen. This is not the place to go into a differential diagnosis between perforating ulcer of the stomach, phlegmonous gastritis, perforating ulcer of the duodenum, obstruction by internal hernia, mesenteric thrombosis, volvulus, acute gangrenous pancreatitis and destructive lesions about the biliary passages. All of these conditions have many symptoms in common and in difficult cases without accurate history it is impossible to come to a definite conclusion as between two or three of them. If one takes too long to study and decide such a case, the patient may die before the conclusion is reached. All of these conditions, however, except mesenteric thrombosis, are surgical, and rather than lose time differentiating between them it would be better to call in immediate surgical aid. In this case the physician should rest content, at least at first, with recognising the gravity of the situation, it being no discredit if one cannot quite decide in the presence of such confusing symptoms; on the other hand, it is discreditable if one fail to appreciate the futility of drugs in such instances.

### THE GALLBLADDER AND DUCTS.

While the ordinary form of catarrhal obstruction of the ducts is doubtless, as held in time past, most commonly due to trouble spreading upward from the duodenum and of evanescent character, it must be remembered that extension of trouble from the liver may also produce it, as well as (which is more to our purpose) carcinoma, gallstones, pancreatitis and hydatids. In cancer of the liver, *e. g.*, jaundice is a very variable sign, depending, perhaps, on an associated catarrh, which may be benefited by treatment while the more serious condition goes unrecognised, at least for a time.

Chronic catarrh of the ducts is associated with dyspeptic symptoms in practically all cases, even of gallstone and cancer, and is frequently the active factor in producing jaundice. It may lead to the production of exceedingly tenacious thick mucus which obstructs the passages, and gives rise to much pain in passing the narrowed channel. Indeed, the suffering of actual gallstone colic can be imitated during the escape of this dense vicious material. In such a case either drainage or cholecystectomy would give relief. In fact, it being impossible to differentiate such an attack from a real gallstone case, and the remedy being alike efficacious in both conditions, no hesitation should be felt in urging operation. Mayo Robson advises gentle irrigation with warm water for a fortnight or so after drainage, in order that the ducts may be the better emptied of their accumulations.

Just as we have an obliterative appendicitis so we may have an obliterative cholecystitis, the gallbladder shrinking to a fraction of its former size, perhaps almost disappearing, being often surrounded by adhesions, giving thereby pain which will be radiated often to one side of the chest or to the other, according as the lower aspect of the liver is involved or the pyloric extremity of the stomach. Here, in this shut sac, no matter how imprisoned, as in the closed appendix or any other shut sac, there is constant production and absorption of toxins of some description, as Dieulafoy has shown, and the degree of toxemia thereby produced may be quite disproportionate. Moreover, the irritation is constant and nagging.

The apparent relation between this catarrh and so-called croupous or membranous enteritis was noted in 1820 by Powell, and recently by Jonathan Hutchinson. Mayo Robson inclines to the view that the cause of the painful attacks followed by slight jaundice is the formation of membrane within the biliary passages, which partially obliterates them, and so sets up spasm of the gallbladder. In fact, complete casts of the gallbladder have been thus formed and passed, in several instances (Mayo Robson, Diseases of the Gallbladder, 3d. Ed. p. 76). If in such cases error in diagnosis should occur and the gallbladder be exposed without the discovery of gallstones, its removal or its drainage would nevertheless be indicated and would give relief.

Bacteria may reach the gallbladder by the ducts, the most common route, through the blood, or by direct passage through its walls, the last being exceptional. Colon and typhoid bacilli are the principal intruders, and both are known to be facultative pyogenic organisms, according to the degree of their virulence and the susceptibility of the patient. Mixed infections may here often be found. Alone or in conjunction with calculi they may produce empyema or fulminating gangrene. A local catarrh by itself is not necessarily followed by empyema, but a catarrh plus bacterial invasion will always occasion serious mischief. Here we have loss of appetite, malaise, local soreness, loss of flesh and chills with fever. This condition has too often in times past been regarded as a combination of malaria and indigestion, in spite of the fact that tenderness is always localised, and is an expression of localised peritonitis. There will often be found a thickening or tumor under the tender area.

The dangers of such a case are somewhat proportionate to its intensity, the greatest of them being perforation and sepsis. Rupture and final escape of pus may occur through the diaphragm, into the liver, into any of the hollow viscera or into the peritoneal cavity.

Aspiration for purposes of diagnosis is exceedingly hazardous; a small opening for exploratory purposes, to be enlarged as circumstances may require, is the life-saving procedure in such a case as this.

When Courvoisier first described gangrenous cholecystitis, in 1890, under the term "phlegmonous inflammation," it was supposed to be an exceedingly rare condition, and he could assemble only seven cases. We now know it as a condition quite analogous to acute gangrenous appendicitis, and have learned that under rarely favoring conditions the gallbladder can go to pieces as rapidly and as totally as that other superfluity, the appendix. Such trouble commences usually with violent pain and vomiting, like most of the acute lesions in the regions under consideration; respiration is rapid and thoracic, depression is marked, tympanitis increases, and there are all the indications of local peritonitis. Jaundice may or may not be present, and fever is an unreliable feature. When gangrene occurs it may be due to thrombosis, bacterial invasion or tension.

Given a due appreciation of the nature and gravity of this condition does it need a surgeon to insist upon the necessity for the promptest possible intervention? Granting even the impossibility of accurate diagnosis should it not be everywhere insisted that such a case is not one for the delay even of an hour's experimentation with drugs? Such a case is essentially one for the surgeon from the hour of the first complaint of pain.

So, too, are even the milder cases of cholangitis, whether it follow chronic pancreatitis, hydatid disease, lumbrici in the ducts, strictures, calculi or malignant disease. These cases are usually characterised by spasmodic pain at intervals, perhaps for years, without jaundice, followed by other spasms, during or after which comes temporary jaundice. Then come attacks of pain followed by chills and perhaps high fever. Tenderness and irregular rigors succeed, and the liver, especially the right lobe, may enlarge, while the gallbladder itself is not usually detected, being often much contracted. The occurrence of chills with slight jaundice and sometimes splenic enlargement will cause this lesion to be frequently mistaken for malaria. The complications most to be dreaded are extension of infection, abscess formation in the liver or gallbladder, involvement of the veins, of the pancreas, or extension of trouble to the thoracic viscera. While the presence of gallstones is the most common cause of suppuration in the ducts, hydatid disease in those parts of the world where it prevails, other parasites, cancer, and the two very common general infections typhoid and influenza, are by no means to be disregarded.

In any condition of this kind, whether acute or chronic, drugs are futile; relief can only be afforded by evacuation and drainage. By this measure septic material is removed, calculi, if present, are taken away, chemosis of mucosa-lined passages is allowed to subside, circulatory equilibrium is restored by relief of pressure, and the kidneys are relieved of the additional burden of excreting biliary material.

Furthermore, and unless conditions are too acute to justify it, adhesions which have both disturbed function and caused referred pains may be separated and general comfort be thus afforded. Perforation of the gallbladder or of the ducts is not so much within the scope of this paper as the conditions which precede and lead to it, but may be mentioned here as one of the dangers to which the physician deliberately exposes a patient when he allows him to go on with vague "dyspeptic" symptoms for which he cannot account.

Intestinal obstruction from gallstones which have escaped is another of these dangers. Acute obstruction from this cause has been considered quite frequent, being placed by Osler as high as one case in thirteen, or by Barnard as one in forty-five cases. Whether such a concretion has gradually made its way along the natural passages, or has produced intense colic and volvulus, or has taken a short course by ulceration, it certainly is fair to say that in every such instance there must have been such frequent or chronic complaint of symptoms referred to the stomach and upper abdomen as to have amply justified exploration.

Of the cystic distensions of the gallbladder, with or without leading symptoms, there are very many cases. In time past it has been a too frequent custom for physicians to treat patients solely on their own descriptions of their ailments, and therefore many an enlarged gallbladder or other suggestive tumor has passed unrecognised for want of careful physical examination. This reproach on the methods of the past will not much longer be deserved; still it cannot yet be quite omitted.

A distended gallbladder is of itself not so much a disease as an indication of its past or present existence. If it be accompanied by absolutely no symptoms it may be regarded as a menace, but perhaps be allowed to remain under observation, with due caution given to the patient. Else, and with the existence of symptoms, it should be regarded as an indication for operation; as much so as a tender enlargement in the region of the appendix. When existent it can usually be detected, though a great deposit of fat or a very rigid and tender abdomen may cause uncertainty. Rarely I have been in such doubt as between it and a floating kidney or a renal tumor that not until exploration was the matter finally settled. In any such case, however, operation of some kind is equally indicated, even though its exact character is only to be determined at the time. Extreme tenderness is one of the great difficulties in making these examinations, but such tenderness in and of itself amply justifies exploration.

Cancer of these parts may be often suspected, but rarely positively recognised until hopeless. The general remarks made elsewhere in this paper may apply here as well. That if recognised reasonably early and if radically removed, the patient may apparently be cured, is well illustrated by a case of my own, a woman about forty years of age, from Lockport, N. Y., who had been under treatment for indigestion and that sort of thing for a long time. When a tumor was finally discovered, she was sent to me. Exploring I found a very large and thick hourglass gallbladder, with a large calculus in each compartment. It was bedded in the liver by a zone of tissue which resembled cancerous tissue, and which the miscroscope later showed to be cancerous. The mass with a goodly portion of apparently normal liver tissue surrounding it was removed, the cautery being used for the purpose. This was nearly four years ago, and the woman is today apparently well, her only complaint being of a ventral hernia. (The opening was large, the patient being corpulent, and large provision for drainage was made.) Such a case as this would seem to justify my claim that cancer is, at least at first, a local disease.

Another feature of considerable importance in cases of calculous disease is enlargement of the liver, especially of the right lobe. When recognised it may be regarded more seriously than it deserves, or it may be a sign of cancerous invasion. In connection with gallstone disease, and particularly with a contracted gallbladder, my attention was first directed to it by my colleague, Dr. Stockton. In many cases it seems to be an evidence solely of hepatic engorgement and in these it quickly subsides after surgical relief of the obstructing cause.

Naturally, the most common of the conditions now under consideration is cholelithiasis. It is estimated that 5 per cent. of mankind have gallstones, from whose presence they may or may not suffer; in some parts of the world the proportion is much larger. With their origin and physical characteristics we have not here to do. But inasmuch as they figure very largely among the causes of intractable dyspepsias they must interest us greatly. That they may occur at any age is not generally enough appreciated. They have even been noted during the first six months of infancy, and between the twentieth and thirtieth year they are far from uncommon. I have had a number of cases, particularly in young women, during this period. At all periods of life they are certainly more common in females. That gallstones may be present in the gallbladder during a large part of a long life and not be recognised is also true.

That they are in many instances the cause or one of the causes of many cases vaguely termed dyspepsia, indigestion, colic, and the like, is now undoubted by those who are well informed, though sufficient stress is not yet placed upon such undeniable facts as to give them their proper importance.

Irregular attacks of paroxysmal pain, often without apparent provocation, radiating from the right hypochondrium or epigastrium toward the right shoulder blade, accompanied or followed by nausea, vomiting or collapse, and followed or not by jaundice, with or without perceptible tumor in the region of the gallbladder, constitute the classical signs of gallstone colic, to which should usually be added a limited abdominal rigidity. In such cases as these diagnosis is not difficult, and one has simply to resign himself to regard the case as surgical.

But it is in cases when symptoms and signs are not classical that both the conscientious and well informed man may easily waver as to whether the stomach, the duodenum, the biliary passages or the pancreas is at fault. Is he to continue to waver, or is he to accept the new teaching that the various lesions as between which he is in doubt are all best recognised and best treated by surgical methods, which, begun as explorations, are concluded by whichever of the expedients now known to surgeons may seem best fitted to their nature and needs?

Cholelithiasis is in effect a surgical disease; temporary relief may be in some cases afforded by internal therapeutics, but this prospect is very vague, and concerns mainly the wealthy and the idle, for whom it is too often fallacious.

The frequency with which gallstones are associated with pyloric stenosis or the old lesions of gastric ulcer constitute an additional reason for operative attack. Furthermore, the frequent association of chronic pancreatitis with stone in the common duct, and the notable relief afforded by removal of the calculus, lends another most forecful argument in favor of operative attack.

In the relatively few (14) years that have elapsed since Courvoisier first successfully removed a stone from the common duct, immense strides in technic have been made, and a well-founded suspicion of calculus anywhere along the biliary passages is now not only sufficient excuse but ample reason for its removal. This is justified not alone by the relief afforded, but by the decreasing mortality rate of the best operators, which is today surprisingly small.

Of operative methods I do not propose to speak; rather of general principles. Personally, I have advised complete removal of the gallbladder, in addition to such further work as may be called for, when this can be accomplished without too greatly complicating the case. I have based this on the facts that it is an essentially superfluous organ in man today, whatever it may have been in past ages in man or animals; that like the appendix and other vestigial remains it is prone to go wrong on slight provocation; that when it does go wrong it becomes a source of offense which has little or no excuse for existence, and consequently that its loss entails no sacrifice but often greatest benefit, and can often be effected without perceptibly enhancing risk. But I certainly would not advise or practise its removal in any instance where this would apparently jeopard the result.

### THE PANCREAS.

The intimate relations between the head of the pancreas and the second part of the duodenum afford an easy explanation for disease traveling in either direction from one to the other, while the peculiar relations between the biliary and pancreatic ducts will account for double or even tripartite lesions. Moreover, the pancreas is easily invaded by ulcer or cancer of the stomach, as well as of the duodenum. Were it my purpose to speak of acute diseases of the pancreas I should say more about the anatomical relations of these parts, in order to indicate how pus may burrow, and to show that practically all diseases of this viscus are surgical in the sense that they are amenable only to surgical relief. Doubtless this will sound like a bold statement to men of the old school, but will be challenged only by those who still believe that the pancreas is an organ of almost unknown function and so placed as to make it sacred from the profaning hands of the surgeon. But inasmuch as I am trying hard to confine myself to those disturbances which upset digestion, and only incidentally or occasionally jeopard life, it will not be necessary to go further in this direction.

It is unquestionably true that the common bile duct is often compressed by the head of the pancreas, by which mild but chronic jaundice may be induced. When this yields to medicinal treatment, as it perhaps may in the beginning, well and good; when it becomes chronic there is no relief save by surgery. Pressure on the pancreas and on its duct, conversely, may be produced during the passage of a gallstone down the common duct; by such pressure again there is more or less obstruction within its subsidiary ducts and backing up of pancreatic secretion. This predisposes it to infection, either from the duodenum or from the infected bile passages, and thus a chronic pancreatitis is produced. In either event a vicious circle is established, and a condition set up which is only relieved by affording physiological rest; *i. e.,* by opening and draining the biliary passages, at the same time removing any calculi which may be met with.

But how are such conditions to be recognised and the indication for surgical intervention made clear?

We must first of all remember that the pancreas is so constructed and protected that it is almost exempt from primary disease. On the other hand, it is peculiarly disposed to secondary involvement which is nearly always an infection,—from the liver and the bile ducts as well as from the duodenum by open highways, or at least highways that once were open from the stomach and from the colon and the abdominal lymphatics by contact or proximity. Mayo Robson has emphasised, moreover, that every function of this organ can be vicariously assumed by some other organ save, perhaps, its glycogenic activity. The stomach can take care of the albuminoids, the salivary and intestinal glands of the starches, and fats can be emulsified by bile and intestinal juices. Furthermore, as in the case of the thyroid, a portion of the organ seems to be as serviceable as the whole, since nearly all of it may be destroyed by necrotic or other morbid process without apparent loss to the patient.

But to return: by what signs and symptoms may we recognise or even suspect pancreatic disease?

Dyspeptic disturbances include anorexia, flatulency (often with offensive eructations), discomfort after eating, nausea, acidity, disrelish for fats and meats. Emaciation is nearly always a feature. The more acute the type of trouble the more marked become the nausea and vomiting. (Of course, in acute pancreatitis "black vomit," *i. e.,* altered blood, is seen earlier than in any other condition). These features should naturally lead to more minute study, not only of the patient, but of the excreta. Naked eye appearances of stools are fallacious. Fat occurs in the stools, as Mayo Robson reminds us (Hunterian Lectures on Diseases of the Pancreas, *London Lancet,* March 19, 1904, *et seq.*) in three forms: as fat droplets, fatty acid crystals and as soap crystals. Moreover, steatorrhea may be met with in jaundice and in enteritis as well; when these coëxist there is much fat in the stools. Consequently the presence of fat in the stools is only pathognomonic when the other conditions are not present, unless there is discovered at the same time a peculiar reaction in the urine, to which I shall allude again in a moment. When, however, there are present in a case evidences of steatorrhea with that faulty digestion of albuminous materials, to which is given the name azotorrhea (undigested muscle fibers, and the like, in the stools), and when the urine contains sugar and gives the peculiar pancreatic reaction, and when especially in addition to these signs any enlargement may be detected in the region of the head of the pancreas, then a positive diagnosis can be made of chronic interstitial pancreatitis.

Hypersecretion of saliva has been noted in some cases of pancreatic disease, it being apparently not only a reflex, but a vicarious act. The so-called pancreatic diarrhea is also significant, when present, it being due to want of digestive power and the propulsion forward of bulky and undigested food. Colorless stools without jaundice should always make one suspicious of pancreatic disease, the absence of color being due not to absence of bile but presence of fat. (Walker, Brunton.)

The pancreas is badly placed for furnishing plain physical signs of disease, especially in stout patients. Nevertheless, it is often possible, especially with the patient in or just removed from the hot bath, and with a warm hand, to feel perceptible enlargement. And often when this cannot be accomplished, one can at least elicit expressions of pain and tenderness which are of themselves significant as indicating something wrong. Further study of such a case will usually indicate that this something would be best more clearly defined and recognised by surgical exploration. In the case of pancreatic calculi tenderness without pain may be elicited.

In the presence of new growths there may be produced on the vena cava such pressure as to cause ascites as well as distension of the gallbladder, or even jaundice by pressure on the hepatic duct. In such cases the mystery can only be solved by exploration. From our point of view, however, no case should be allowed to go on to such an extent without it.

Another feature of interest in some of these cases is tendency to general hemorrhage from all exposed raw or mucous surfaces as well as from vascular ruptures into organs and tissues, particularly into the pancreas itself (pancreatic apoplexy). Of course, this is due to alterations in the blood, not yet sufficiently studied, but certainly in part characterised by diminution in the number of blood plates and by enfeebled coagulability. In this condition, particularly emphasised by him, Mayo Robson's advice to give large doses of calcium chloride (30 to 60 grains) three or four times daily, should be followed, since this salt has a marked effect in increasing coagulability.

So much has been already said about jaundice as an indication for operation in all chronic and many acute cases that it is hardly necessary here to point out its significance when pancreatic disease is suspected. Glycosuria is a matter of even greater importance. When present it is to be interpreted as meaning that the islands of Langerhans are involved, *i. e.,* that the pancreatic lesion is of interstitial and interacinous type; whereas in disease of rather the interlobular type these islands are not encroached upon, at least not until late, and so they escape. Glycosuria, then, is significant when detected, but its absence gives only negative value to the test. So too in cancer, when only a part of the pancreas is involved it will be absent.

Much is to be hoped for from a new reaction which Mr. Cammidge has but recently described (The Chemistry of the Urine in Diseases of the Pancreas, *London Lancet,* March 19, 1904,) but has been putting to practical test for some years, and in which our best authority, Mayo Robson, seems now to have quite implicit confidence. By means of this he seems to be able to determine as between malignant and nonmalignant pancreatic disease, and thereby to have greatly assisted the surgeon, since many conditions suspected to be cancerous can thereby be shown to be nonmalignant, and the surgeon's sphere of usefulness thereby enlarged. This test is too complicated to be here described, but my personal acquaintance with and confidence in both Mr. Mayo Robson and Mr. Cammidge, incline me to feel that they have largely added to our resources by its introduction.

Of pancreatic cancer, cysts and calculi, I hardly need speak here in detail. Whether certainly recognised or sincerely suspected they should alike be given the chance which only the surgeon's knife affords.

But it is right to stop and answer the question surely prompted by the above statements. What can surgery offer in these rather numerous cases of chronic pancreatitis and allied conditions?

First of all, exact diagnosis, and this will of itself afford great comfort though the condition revealed be inoperable.

Next, direct access can be made to not only the biliary passages, but to the head of the pancreas and its ducts, if necessary, by opening the duodenum and directly exploring the duct of Wirsung, or for the removal of calculi. In the majority of cases, however, it will be found necessary and at the same time sufficient to open and drain the common duct, of course removing any calculi that may be discovered, and not resting until a probe has been passed completely through it and its patulency reëstablished and demonstrated. In a goodly proportion of cases it will prove sufficient to do a cholecystotomy by which there shall be afforded opportunity for drainage and antiseptic irrigation. At other times a cholecystduodenostomy will be indicated, by which bile may be more easily poured into the bowel. Behind all these procedures there are the underlying motives of affording not only direct outpour of obstructed secretions, but physiological rest. After their performance appetite returns, digestion is resumed, body weight increases, jaundice disappears, pain and tenderness subside, cachexia disappears, and for a condition of general ill-health is substituted one of vigor and general welfare which no amount of drugs or time spent in dietetic "cures" could accomplish. True, this is effected at the expense of operation with its attendant dangers. In the hands of our best surgeons these risks are now so small that they should cut no very large figure in deciding as to the best course; nevertheless, they can never be left out of account.

In conclusion I would summarise thus: operation is indicated in:

Gastric ulcer.—Either gastrotomy with direct attack upon the involved surface, or gastroenterostomy (posterior), to afford rest and a more direct outlet.

Gastric dilatation.—When lavage and the customary internal measures have proved disappointing, and always when pylorostenosis exists.

Gastric cancer.—Early if possible, hoping to effect a cure; when late it may be still possible to make a gastroenterostomy which shall prolong life. In the most advanced cases, one may have to be content with a jejunostomy for temporary purposes.

Gastric anomalies.—Such as hour glass stomach, and the like.

Pyloric obstruction.—Here one must choose as between excision with end to end reunion, pyloroplasty, or anastomosis, preferably posterior.

Duodenal ulcer.—Here a posterior gastroenterostomy is clearly indicated.

Duodenal cancer and duodenal stricture.—Of these also the same is true.

Biliary obstruction.—All chronic and many acute cases, whether due to intrinsic or extrinsic causes (cholangitis, gallstones, cancer, old adhesions.) In these some operative intervention is imperative; whether it shall consist of extirpation, opening and drainage, or anastomosis, depending on the conditions revealed through the exploratory incision.

Pancreatic disease of almost every type, whether acute or chronic. In the former, posterior drainage must usually be added to the attack from the front. In the latter, it will usually suffice to open and drain the biliary passages; rarely a cholecystenterostomy may be called for.

Finally, in all cases of acute pain in the upper abdomen accompanied or followed by vomiting, especially of recent or old blood, by tympanitis, muscle spasm and collapse, and in all chronic cases of pain and tenderness in the region described, with a history of recent and particularly of long standing symptoms, of emaciation, dyspepsia and indigestion, with or without perceptible tumor, but with rigidity of the rectus and other abdominal muscles,—in all of these, I say, I would urge surgical exploration, after due preparation, and under circumstances which may allow the surgeon to resort to every technical and life-saving expedient known to the art. This advice is given as the result of convictions that steadily strengthen as experience accumulates and with a sincere belief that when it becomes general routine, as it is sure to in time, the greatest good will be done to the greatest number.

510 Delaware Avenue.

